# 3,6-Diiodo-9*H*-carbazole

**DOI:** 10.1107/S1600536812012901

**Published:** 2012-03-31

**Authors:** Yu-Zhong Xie, Jing-Yi Jin, Guang-De Jin

**Affiliations:** a Department of Chemistry, Yanbian University, Yanji Jilin 133002, People’s Republic of China

## Abstract

In the title compound, C_12_H_7_I_2_N, the tricyclic aromatic ring system is essentially planar, with an r.m.s. deviation of 0.0272 Å. The two I atoms are marginally out of plane, with the C—I bonds angled at 3.9 (2) and 1.1 (2)° with respect to the planes of their respective benzene rings, above and below the plane of the carbazole ring system. No classical hydrogen bonds are observed in the crystal structure.

## Related literature

For the synthesis of the title compound, see: Tucker (1926 ▶); Lengvinaite *et al.* (2007 ▶). For related compounds, see: Grigalevicius *et al.* (2007 ▶); Cui *et al.* (2009 ▶); Tian *et al.* (2010 ▶); Klejevskaja *et al.* (2007 ▶). For their applications, see: Zhang *et al.* (2009 ▶); Zhong’an *et al.* (2010 ▶); Lu *et al.* (2006 ▶); Grigalev­icius *et al.* (2006 ▶, 2011 ▶).
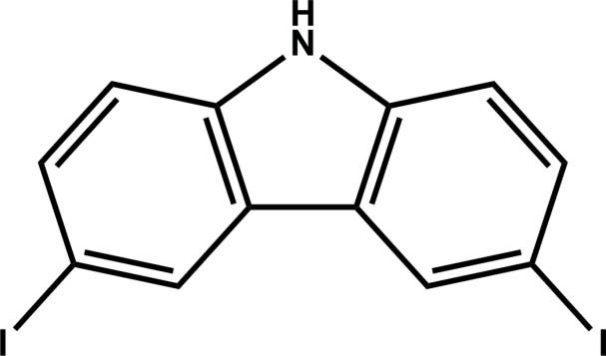


## Experimental

### 

#### Crystal data

C_12_H_7_I_2_N
*M* *_r_* = 418.99
Orthorhombic, 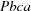

*a* = 11.8823 (14) Å
*b* = 7.8835 (9) Å
*c* = 24.835 (3) Å
*V* = 2326.4 (5) Å^3^
*Z* = 8
Mo *K*α radiation
μ = 5.37 mm^−1^
*T* = 293 K
0.23 × 0.21 × 0.18 mm

#### Data collection

Bruker APEXII CCD area-detector diffractometer
Absorption correction: multi-scan (*SADABS*; Bruker, 2004 ▶) *T*_min_ = 0.371, *T*_max_ = 0.445
12303 measured reflections
2456 independent reflections
1879 reflections with *I* > 2σ(*I*)
*R*_int_ = 0.032

#### Refinement

*R*[*F* ^2^ > 2σ(*F* ^2^)] = 0.026
*wR*(*F* ^2^) = 0.052
*S* = 1.05
2456 reflections
141 parameters
H atoms treated by a mixture of independent and constrained refinement
Δρ_max_ = 0.57 e Å^−3^
Δρ_min_ = −0.56 e Å^−3^

### 

Data collection: *APEX2* (Bruker, 2004 ▶); cell refinement: *SAINT-Plus* (Bruker, 2004 ▶); data reduction: *SAINT-Plus*; program(s) used to solve structure: *SHELXS97* (Sheldrick, 2008 ▶); program(s) used to refine structure: *SHELXL97* (Sheldrick, 2008 ▶); molecular graphics: *XP* in *SHELXTL* (Sheldrick, 2008 ▶); software used to prepare material for publication: *SHELXTL*.

## Supplementary Material

Crystal structure: contains datablock(s) global, I. DOI: 10.1107/S1600536812012901/pk2397sup1.cif

Supplementary material file. DOI: 10.1107/S1600536812012901/pk2397Isup2.cdx

Structure factors: contains datablock(s) I. DOI: 10.1107/S1600536812012901/pk2397Isup3.hkl

Supplementary material file. DOI: 10.1107/S1600536812012901/pk2397Isup4.cml

Additional supplementary materials:  crystallographic information; 3D view; checkCIF report

## Figures and Tables

**Fig. 1.**
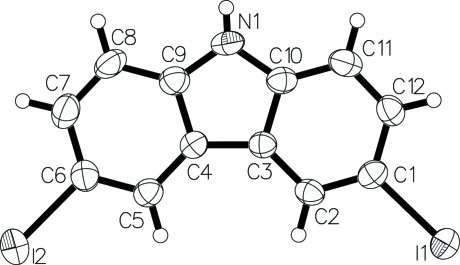
The molecular structure of the title coumpound. Displacement ellipsoids are drawn at 30% probability level..

## supplementary crystallographic information

### Comment

Carbazole moieties are an important construction block for hole-transporting and electroluminescent materials. 3,6-diiodo-9*H*-carbazole has been used to design and synthesize carbazole derivatives. We report herein the crystal structure of the title compound. The molecular structure is shown in Fig. 1. All bond lengths and angles are within normal ranges. The tricyclic aromatic ring system is essentially planar with an r.m.s. deviation of 0.0272 Å. There are no classical hydrogen bonds observed in the crystal structure.

### Experimental

The title compound was synthesized according to a literature method (Tucker, 1926). Colorless crystals were obtained from a solution in chloroform upon slow evaporation of the solvent.

### Refinement

The nitrogen-bound H atom was located in a difference Fourier map and refined freely (N—H = 0.75 (3) Å). Carbon-bound H atoms were positioned geometrically (C—H = 0.93 Å) and refined using a riding model, with *U*_iso_(H) = 1.2 U_eq_(C).

### Crystal data

**Table:**

C_12_H_7_I_2_N	*F*(000) = 1536
*M**_r_* = 418.99	*D*_x_ = 2.392 Mg m^−^^3^
Orthorhombic, *P**b**c**a*	Mo *K*α radiation, λ = 0.71073 Å
Hall symbol: -P 2ac 2ab	Cell parameters from 12303 reflections
*a* = 11.8823 (14) Å	θ = 1.6–26.8°
*b* = 7.8835 (9) Å	µ = 5.37 mm^−^^1^
*c* = 24.835 (3) Å	*T* = 293 K
*V* = 2326.4 (5) Å^3^	Block, colorless
*Z* = 8	0.23 × 0.21 × 0.18 mm

### Data collection

**Table:**

Bruker APEXII CCD area-detector diffractometer	2456 independent reflections
Radiation source: fine-focus sealed tube	1879 reflections with *I* > 2σ(*I*)
Graphite monochromator	*R*_int_ = 0.032
φ and ω scans	θ_max_ = 26.8°, θ_min_ = 1.6°
Absorption correction: multi-scan (*SADABS*; Bruker, 2004)	*h* = −8→15
*T*_min_ = 0.371, *T*_max_ = 0.445	*k* = −9→9
12303 measured reflections	*l* = −31→29

### Refinement

**Table:**

Refinement on *F*^2^	Secondary atom site location: difference Fourier map
Least-squares matrix: full	Hydrogen site location: inferred from neighbouring sites
*R*[*F*^2^ > 2σ(*F*^2^)] = 0.026	H atoms treated by a mixture of independent and constrained refinement
*wR*(*F*^2^) = 0.052	*w* = 1/[σ^2^(*F*_o_^2^) + (0.0193*P*)^2^ + 0.8116*P*] where *P* = (*F*_o_^2^ + 2*F*_c_^2^)/3
*S* = 1.05	(Δ/σ)_max_ = 0.002
2456 reflections	Δρ_max_ = 0.57 e Å^−^^3^
141 parameters	Δρ_min_ = −0.56 e Å^−^^3^
0 restraints	Extinction correction: *SHELXL97* (Sheldrick, 2008), Fc^*^=kFc[1+0.001xFc^2^λ^3^/sin(2θ)]^-1/4^
Primary atom site location: structure-invariant direct methods	Extinction coefficient: 0.00021 (4)

### Special details

**Table:**

Geometry. All e.s.d.'s (except the e.s.d. in the dihedral angle between two l.s. planes) are estimated using the full covariance matrix. The cell e.s.d.'s are taken into account individually in the estimation of e.s.d.'s in distances, angles and torsion angles; correlations between e.s.d.'s in cell parameters are only used when they are defined by crystal symmetry. An approximate (isotropic) treatment of cell e.s.d.'s is used for estimating e.s.d.'s involving l.s. planes.
Refinement. Refinement of *F*^2^ against ALL reflections. The weighted *R*-factor *wR* and goodness of fit *S* are based on *F*^2^, conventional *R*-factors *R* are based on *F*, with *F* set to zero for negative *F*^2^. The threshold expression of *F*^2^ > σ(*F*^2^) is used only for calculating *R*-factors(gt) *etc*. and is not relevant to the choice of reflections for refinement. *R*-factors based on *F*^2^ are statistically about twice as large as those based on *F*, and *R*- factors based on ALL data will be even larger.

### Fractional atomic coordinates and isotropic or equivalent isotropic displacement parameters (Å^2^)

**Table:**

	*x*	*y*	*z*	*U*_iso_*/*U*_eq_	
C11	0.7583 (3)	0.4740 (4)	0.77946 (15)	0.0445 (9)	
H11	0.8290	0.5240	0.7822	0.053*	
C12	0.6978 (3)	0.4308 (4)	0.82476 (14)	0.0406 (9)	
H12	0.7277	0.4525	0.8587	0.049*	
H1N	0.807 (3)	0.511 (4)	0.6716 (13)	0.032 (11)*	
I2	0.35650 (2)	0.22435 (4)	0.536173 (10)	0.06040 (11)	
I1	0.50690 (2)	0.28678 (4)	0.891514 (9)	0.04915 (10)	
N1	0.7525 (3)	0.4684 (4)	0.67876 (13)	0.0460 (8)	
C5	0.4904 (3)	0.2882 (4)	0.63769 (13)	0.0387 (8)	
H5	0.4264	0.2467	0.6548	0.046*	
C4	0.5818 (3)	0.3460 (4)	0.66751 (12)	0.0333 (8)	
C6	0.4967 (3)	0.2939 (5)	0.58238 (14)	0.0432 (9)	
C9	0.6766 (3)	0.4125 (4)	0.64077 (14)	0.0402 (9)	
C1	0.5918 (3)	0.3545 (4)	0.82033 (13)	0.0373 (8)	
C2	0.5437 (3)	0.3208 (4)	0.77121 (13)	0.0357 (8)	
H2	0.4730	0.2705	0.7690	0.043*	
C3	0.6032 (3)	0.3638 (4)	0.72449 (12)	0.0333 (8)	
C8	0.6826 (3)	0.4177 (5)	0.58496 (14)	0.0478 (10)	
H8	0.7461	0.4603	0.5677	0.057*	
C7	0.5931 (3)	0.3587 (5)	0.55598 (14)	0.0492 (10)	
H7	0.5954	0.3612	0.5186	0.059*	
C10	0.7105 (3)	0.4408 (4)	0.72961 (14)	0.0375 (8)	

### Atomic displacement parameters (Å^2^)

**Table:**

	*U*^11^	*U*^22^	*U*^33^	*U*^12^	*U*^13^	*U*^23^
C11	0.033 (2)	0.047 (2)	0.054 (2)	−0.0035 (18)	0.0000 (19)	−0.0083 (18)
C12	0.040 (2)	0.043 (2)	0.0391 (19)	0.0011 (17)	−0.0046 (17)	−0.0074 (16)
I2	0.0586 (2)	0.0815 (2)	0.04106 (16)	−0.00842 (15)	−0.01063 (13)	0.00035 (13)
I1	0.04885 (17)	0.06034 (18)	0.03825 (14)	−0.00679 (13)	0.00108 (12)	0.00815 (11)
N1	0.0340 (19)	0.055 (2)	0.0490 (18)	−0.0110 (17)	0.0093 (18)	0.0011 (16)
C5	0.036 (2)	0.040 (2)	0.039 (2)	0.0034 (17)	0.0027 (17)	0.0040 (16)
C4	0.034 (2)	0.0312 (18)	0.0345 (18)	0.0029 (15)	0.0017 (16)	0.0029 (14)
C6	0.044 (2)	0.048 (2)	0.0375 (19)	−0.0003 (19)	−0.0025 (17)	−0.0026 (17)
C9	0.036 (2)	0.043 (2)	0.042 (2)	0.0021 (17)	0.0030 (17)	0.0004 (16)
C1	0.038 (2)	0.0355 (19)	0.0385 (18)	0.0031 (17)	0.0052 (17)	0.0040 (16)
C2	0.0298 (18)	0.034 (2)	0.0427 (19)	−0.0003 (15)	−0.0044 (16)	0.0050 (15)
C3	0.0312 (19)	0.0328 (19)	0.0358 (18)	0.0010 (15)	0.0002 (15)	0.0010 (14)
C8	0.041 (2)	0.061 (3)	0.041 (2)	0.003 (2)	0.0151 (18)	0.0017 (18)
C7	0.055 (3)	0.059 (3)	0.0341 (19)	0.005 (2)	0.0050 (19)	−0.0020 (17)
C10	0.0331 (19)	0.037 (2)	0.043 (2)	0.0003 (16)	0.0043 (17)	−0.0027 (16)

### Geometric parameters (Å, º)

**Table:**

C11—C12	1.378 (5)	C5—H5	0.9300
C11—C10	1.387 (5)	C4—C9	1.409 (5)
C11—H11	0.9300	C4—C3	1.445 (4)
C12—C1	1.400 (5)	C6—C7	1.415 (5)
C12—H12	0.9300	C9—C8	1.389 (5)
I2—C6	2.096 (4)	C1—C2	1.373 (4)
I1—C1	2.104 (3)	C2—C3	1.400 (4)
N1—C10	1.375 (4)	C2—H2	0.9300
N1—C9	1.378 (5)	C3—C10	1.418 (5)
N1—H1N	0.75 (3)	C8—C7	1.366 (5)
C5—C6	1.376 (5)	C8—H8	0.9300
C5—C4	1.391 (5)	C7—H7	0.9300
			
C12—C11—C10	117.9 (3)	C8—C9—C4	121.5 (3)
C12—C11—H11	121.0	C2—C1—C12	121.8 (3)
C10—C11—H11	121.0	C2—C1—I1	119.8 (3)
C11—C12—C1	120.8 (3)	C12—C1—I1	118.3 (3)
C11—C12—H12	119.6	C1—C2—C3	118.6 (3)
C1—C12—H12	119.6	C1—C2—H2	120.7
C10—N1—C9	109.9 (3)	C3—C2—H2	120.7
C10—N1—H1N	127 (3)	C2—C3—C10	118.9 (3)
C9—N1—H1N	123 (3)	C2—C3—C4	134.4 (3)
C6—C5—C4	118.5 (3)	C10—C3—C4	106.7 (3)
C6—C5—H5	120.7	C7—C8—C9	118.4 (4)
C4—C5—H5	120.7	C7—C8—H8	120.8
C5—C4—C9	119.7 (3)	C9—C8—H8	120.8
C5—C4—C3	133.7 (3)	C8—C7—C6	120.6 (3)
C9—C4—C3	106.6 (3)	C8—C7—H7	119.7
C5—C6—C7	121.2 (3)	C6—C7—H7	119.7
C5—C6—I2	119.6 (3)	N1—C10—C11	129.9 (3)
C7—C6—I2	118.9 (3)	N1—C10—C3	108.2 (3)
N1—C9—C8	129.8 (3)	C11—C10—C3	121.9 (3)
N1—C9—C4	108.7 (3)		
			
C10—C11—C12—C1	0.5 (5)	C5—C4—C3—C2	4.6 (7)
C6—C5—C4—C9	1.7 (5)	C9—C4—C3—C2	−179.0 (4)
C6—C5—C4—C3	177.7 (4)	C5—C4—C3—C10	−176.4 (4)
C4—C5—C6—C7	−0.8 (5)	C9—C4—C3—C10	0.1 (4)
C4—C5—C6—I2	−175.7 (2)	N1—C9—C8—C7	−177.6 (4)
C10—N1—C9—C8	178.7 (4)	C4—C9—C8—C7	1.0 (5)
C10—N1—C9—C4	0.0 (4)	C9—C8—C7—C6	−0.1 (6)
C5—C4—C9—N1	177.0 (3)	C5—C6—C7—C8	0.0 (6)
C3—C4—C9—N1	0.0 (4)	I2—C6—C7—C8	174.9 (3)
C5—C4—C9—C8	−1.8 (5)	C9—N1—C10—C11	178.8 (4)
C3—C4—C9—C8	−178.8 (3)	C9—N1—C10—C3	0.0 (4)
C11—C12—C1—C2	−0.4 (5)	C12—C11—C10—N1	−179.0 (4)
C11—C12—C1—I1	178.5 (3)	C12—C11—C10—C3	−0.4 (5)
C12—C1—C2—C3	0.3 (5)	C2—C3—C10—N1	179.1 (3)
I1—C1—C2—C3	−178.6 (2)	C4—C3—C10—N1	−0.1 (4)
C1—C2—C3—C10	−0.2 (5)	C2—C3—C10—C11	0.3 (5)
C1—C2—C3—C4	178.7 (4)	C4—C3—C10—C11	−178.9 (3)

## References

[bb1] Bruker (2004). *APEX2*, *SAINT-Plus* and *SADABS* Bruker AXS Inc., Madison, Wisconsin, USA.

[bb2] Cui, J., Duan, M. & Cai, L. (2009). *Acta Cryst.* E**65**, o216. 10.1107/S1600536808042827 PMC2968375 21581835

[bb3] Grigalevicius, S., Ma, L., Grazulevicius, J. V. & Xie, Z. (2006). *Synth. Met.* **156**, 46–50.

[bb4] Grigalevicius, S., Ma, L., Qian, G., Xie, Z., Forster, M. & Scherf, U. (2007). *Macromol. Chem. Phys.* **208**, 349–355.

[bb5] Grigalevicius, S., Zhang, B., Xie, Z., Forster, M. & Scherf, U. (2011). *Org. Electron.* **12**, 2253–2257.

[bb6] Klejevskaja, B., Burbulis, E., Michaleviciute, A., Ostrauskaite, J., Grazuleviius, J. V. & Jankauskas, V. (2007). *Synth. Met.* **157**, 968–973.

[bb7] Lengvinaite, S., Grazulevicius, J. V., Jankauskas, V. & Grigalevicius, S. (2007). *Synth. Met.* **157**, 529–533.

[bb8] Lu, J., Xia, P. F., Lo, P. K., Tao, Y. & Wong, M. S. (2006). *Chem. Mater.* **18**, 6194–6203.

[bb9] Sheldrick, G. M. (2008). *Acta Cryst.* A**64**, 112–122. 10.1107/S0108767307043930 18156677

[bb10] Tian, N., Lenkeit, D., Pelz, S., Fischer, L. H., Escudero, D., Schiewek, R., Klink, D., Schmitz, O. J., Gonzalez, L., Schaferling, M. & Holder, E. (2010). *Eur. J. Inorg. Chem.* pp. 4875–4885.

[bb11] Tucker, S. H. (1926). *J. Chem. Soc.* pp. 546–553.

[bb12] Zhang, H., Wang, S., Li, Y., Zhang, B., Du, C., Wan, X. & Chen, Y. (2009). *Tetrahedron*, **65**, 4455–4463.

[bb13] Zhong’an, L., Zuoquan, J., Guofu, Q., Wenbo, W., Gui, Y., Yunqi, L., Jingui, Q. & Zhen, L. (2010). *Macromol. Chem. Phys.* **211**, 1820–1825.

